# Characterization of a *de novo GABBR2* variant linked to autism spectrum disorder

**DOI:** 10.3389/fnmol.2023.1267343

**Published:** 2023-11-23

**Authors:** Noa Bielopolski, Michal Stawarski, Ilana Roitman, Karen Fridman, Shane Wald-Altman, Simon Früh, Bernhard Bettler, Andreea Nissenkorn

**Affiliations:** ^1^QR Genetics, Tel Aviv, Israel; ^2^Department of Biomedicine, Pharmazentrum, University of Basel, Basel, Switzerland; ^3^Pediatric Neurology Unit, Edith Wolfson Medical Center, Holon, Israel; ^4^Magen Center for Rare Diseases, Edith Wolfson Medical Center, Holon, Israel; ^5^Department of Pediatrics, The School of Medicine, Tel Aviv University, Tel Aviv, Israel

**Keywords:** GABA_B_ receptor, autism, molecular dynamics, VUS classification, functional assay

## Abstract

GABA_B_ receptors (GABA_B_Rs) are G protein-coupled receptors for γ-aminobutyric acid (GABA), the major inhibitory neurotransmitter in the central nervous system. Pathogenic variants in the *GABBR1* and *GABBR2* genes, which encode the GB1 and GB2 subunits of GABA_B_Rs, are implicated in several neurological and developmental disorders, including epilepsy and autism. Here we present a 7-year-old boy with Level 3 Autism Spectrum Disorder who carries a *de novo* heterozygous missense *GABBR2* p.Arg212Gln variant. This variant was identified through whole exome sequencing and classified as variant of unknown significance (VUS). Treatment with the GABA_B_R agonist baclofen showed no clinical improvement, raising the question whether this VUS is responsible for the patient’s phenotype. We conducted a study to investigate the impact of the *GABBR2* p.Arg212Gln and the previously reported *GABBR2* p.Arg212Trp variants on protein structure and receptor activity. This study utilized a combination of molecular dynamics (MD) simulations, and *in vitro* experiments. Our simulations demonstrate that both amino acid substitutions locally alter amino acid interactions in the extracellular domain of GB2. Most importantly, the substitutions influence the positioning of transmembrane helices, shifting the conformation towards an active state with *GABBR2* p.Arg212Gln and an inactive state with *GABBR2* p.Arg212Trp. Functional assays confirmed the MD predictions, as evidenced by increased constitutive activity and enhanced potency of GABA for *GABBR2* p.Arg212Gln, and a decreased constitutive activity with a loss of GABA potency for *GABBR2* p.Arg212Trp. Our findings demonstrate the utility of MD simulations in predicting the functional consequences of VUS. Clarifying the pathogenic mechanisms associated with gene variants will aid in the identification of personalized treatment approaches.

## Introduction

GABA_B_ receptors (GABA_B_Rs) are G protein-coupled receptors for the neurotransmitter γ-aminobutyric acid (GABA). They play a crucial role in regulating neurotransmitter release and neuronal inhibition by reducing cAMP levels and gating Ca^2+^ and K^+^ channels (Pin and Bettler, 2016). GABA_B_Rs are heterodimers composed of a GB1 subunit paired with a GB2 subunit (GB1/2 receptors). GB1 and GB2 subunits consist of three domains: an extracellular Venus-fly-trap domain (VFTD), a heptahelical transmembrane domain (7TMD), and a C-terminal intracellular domain. The VFTD of the GB1 subunit contains the orthosteric binding site for GABA while the GB2 subunit couples with the G protein. The VFT module of each GABA_B_R subunit contains two domains or lobes, LB1 and LB2, with LB1 resting atop LB2 and reaching further into the extracellular space. Only fully assembled heterodimers exit the endoplasmic reticulum and traffic to the cell surface.

GB1 and GB2 receptor subunits are encoded by the *GABBR1* and *GABBR2* genes, respectively. Several *de novo* pathogenic variants in both *GABBR1* (Cediel et al., 2022) and *GABBR2* (Lopes et al., 2016; Yoo et al., 2017; Vuillaume et al., 2018; Samanta and Zarate, 2019; D’Onofrio et al., 2022) have been described. Known pathogenic *GABBR1* variants display a loss-of-function, whereas *GABBR2* variants typically exhibit increased constitutive receptor activity and reduced EC50, indicative of a gain-of-function. However, gain-of-function *GABBR2* variants also reduce maximal activity (Emax) in functional assay systems, which opposes the gain-of-function effects (Yoo et al., 2017).

Structures of the GABA_B_R heterodimer in the apo, antagonist-bound, agonist-bound and agonist/PAM-bound states in complex with the G protein have been resolved at high resolution (Mao et al., 2020; Papasergi-Scott et al., 2020; Park et al., 2020; Shaye et al., 2020, 2021; Shen et al., 2021). These structures provide a detailed understanding of the activation mechanism. GABA binding to the orthosteric binding site in the VFTD of the GB1 subunit induces closure of the VFTD and compaction of the VFTD interface. This conformational change propagates to the 7TMDs, resulting in the disruption of intersubunit ionic locks between TM3 and TM6 that maintain the 7TMDs in an inactive state (Papasergi-Scott et al., 2020; Park et al., 2020; Shaye et al., 2020, 2021). A new helical interface forms along the two TM6 domains (Xue et al., 2019; Shaye et al., 2020), ultimately activating the G protein at GB2. Single-molecule FRET studies revealed that VFTD dimers oscillate between active and inactive orientations in the absence of agonists (Olofsson et al., 2014), which likely underlies constitutive activity of the receptor (Grünewald et al., 2002; Rajalu et al., 2015).

Pathogenic variants in *GABBR2* have been associated with various neurological phenotypes, including epileptic encephalopathy, intellectual disability, autism, and Rett-like features (Yoo et al., 2017; Vuillaume et al., 2018; Samanta and Zarate, 2019). Detailed *in vitro* (Yoo et al., 2017; Vuillaume et al., 2018) and *in vivo* (Yoo et al., 2017) studies revealed functional alterations caused by pathogenic variants in the *GABBR2* gene (Yoo et al., 2017). However, functional assay systems may not always be available to clinicians. Advanced computational methods, such as molecular dynamics (MD), offer insights into protein–protein interactions and can help predict structural changes. With the availability of experimental 3D structures, *in silico* methods have been employed to investigate various receptor features, including conformational changes in the extracellular domain and ligand binding (Melis et al., 2006, 2008; Geitmann et al., 2010; Ashby et al., 2012), as well as characterization of drug binding sites (Sanghvi et al., 2008). Some of these *in silico* findings are supported by functional data. For example, in the GABA_A_-rho receptor (formerly known as the GABA_C_ receptor), MD simulations revealed the importance of a loop stabilizing GABA’s carboxylate, a finding later confirmed by mutagenesis studies (Melis et al., 2008).

Here, we used MD simulations and our in-house algorithms to examine and characterize the spatial alterations induced by two missense variants of unknown significance (VUS) occurring at the same position in the *GABBR2* gene: the p.Arg212Gln variant identified in our patient and the p.Arg212Trp variant documented earlier in gnomAD.^1^ Our MD simulations revealed alterations in both the VFTD, where the variants are located, and the 7TMDs. MD simulations predict that the p.Arg212Gln variant of the GB2 subunit (GB2^R212Q^) favors the active state of the receptor, whereas the p.Arg212Trp variant of GB2 (GB2^R212W^) favors the inactive state of the receptor. Functional analysis reveals that GB2^R212Q^ increases constitutive receptor activity, enhances potency of GABA, and reduces Emax, while GB2^R212W^ reduces constitutive activity and potency of GABA. These findings highlight how variants in the extracellular domain influence the TM domains and shed light on how structural changes impact GABA_B_R activity. Importantly, this study not only provides insights into specific variants and their effects but also offers a potential avenue for clinicians to evaluate and treat patients with VUS in the *GABBR2* gene.

## Materials and methods

### Case presentation

The index case is a 7-year-old boy treated at our Center for Rare Diseases for developmental encephalopathy and autistic spectrum disorder (ASD). He was born by Caesarian section at 41 weeks after an uneventful pregnancy and delivery to healthy unrelated parents. Global developmental delay was apparent from the first year of life, with the patient starting to walk at 2 years of age but never acquiring speech. At the age of two, repeated staring spells prompted an EEG examination that revealed multifocal polyspikes. Following a video-EEG evaluation, these events were considered non-epileptic, and therefore, anti-epileptic treatment was not initiated. By the age of three, the EEG normalized. Brain MRI results were normal. The patient also developed severe gastrointestinal symptoms, which were attributed to eosinophilic gastroenteritis and partially responded to steroid treatment. At 3 years of age, the patient was non-verbal, lacked eye contact, and displayed extreme hyperactive behavior and self-inflicted injuries. Additional symptoms included bruxism (teeth grinding) and hyperventilation episodes, resembling a Rett-like phenotype. He was diagnosed with level 3 Autistic Spectrum Disorder and was referred for genetic diagnosis. Whole exome trio sequencing revealed a *de novo* heterozygous VUS, c.635G > A, p.Arg212Gln in *GABBR2*. In an attempt to address the patient’s condition, treatment with the GABA_B_R agonist baclofen at a dosage of 0.7 mg/kg was administered, but no improvement was observed. Due to the lack of response to treatment, the pathogenicity of the *GABBR2* variant and its association with the patient’s phenotype came into question. To assess the clinical relevance of the *GABBR2* p.Arg212Gln variant, the parents sought the services of QR Genetics Ltd. for MD simulation and analysis. The p.Arg212Gln variant was additionally analyzed in an established functional assay system in heterologous cells to validate the MD simulation. We also analyzed the *GABBR2* p.Arg212Trp variant that is listed as a VUS in gnomAD (see footnote 1). The legal guardians (parents) signed a written consent to participate in this publication. This report was approved by the local Ethics Committee at the Wolfson Medical Center and was exempt from an IRB protocol as a case report.

### Whole exome sequencing

Trio whole-exome sequencing was conducted on the patient’s and parents’ DNA at the Otogenetics CLIA laboratories. The samples were enriched using the 51 MB Agilent Human All Exon V5 kit 51 Mb. Sequencing was performed on the Illumina HiSeq 2,500 platform with 125-bp paired-end runs, resulting in an average depth coverage of 89. The sequencing reads were aligned to the reference GRCh37/hg19 genome. The FASTQ data, certified by EMQN, was transferred to the Hadassah Molecular Lab for analysis. Dataset files information were processed with the following filtering steps: Frequent variants (MAF > 0.1% in Gnomad), intronic variants more than 8 bp from the intron-exon boundary, and synonymous variants (except those within canonical splicing sites) were filtered out. Variants were annotated according to OMIM genes and pathogenic prediction tools. No relevant homozygous, hemizygous, or double heterozygous variants were identified. However, one heterozygous variant in *GABBR2* (NM_005458.8), located at chr9:101258792, c.635G > A, resulting in the p.Arg212Gln amino acid change, was found to be *de novo* in the patient and assessed as a VUS following ACMG guidelines.

### Preparation of receptor for MD

The atomic coordinates of the protein crystal structures of human GABA_B_R (PDB ID-7 EB2, 7C7S) were downloaded from the RCSB-PDB (protein data bank) database. The complex was prepared using protein preparation wizard in Maestro 12.0 (Maestro v9.2. Portland, OR: Schrödinger, Inc.; 2011) for both the wild type (GB2 WT) and mutated *GABBR2* p.Arg212Gln protein and the antagonist CGP54626 was removed from PDB-7C7S. H-bond network optimization was carried out assuming a neutral pH of the solution, followed by water molecules removal. An all-atom minimization step was carried out to remove unfavorable steric clashes until a convergence was reached or with a maximum RMSD of 0.3 Å from the original conformation using force field module OPLS3e. No steric clashes were reported after the final minimization step (O’Boyle et al., 2011; Chandel et al., 2020; Kumar et al., 2020; Raj et al., 2020).

### MD simulation

For MD simulations, systems were built for GABA_B_Rs using the system builder panel of Desmond (Schrödinger Release 2022-2). The SPC solvent model was used, and the force field was set to OPLS4e. Membrane was placed using OPM.^2^ The protein was inserted into an orthorhombic box with buffer dimensions 10 × 10 × 10 Å^3^ and NPγT ensemble. The total simulation time for each system was set at 250 ns. Simulations were set to run at 300.0 K and at 1.01325 bar. The option to relax model systems before simulations was selected.

### MD analysis

Trajectories were analyzed by MDAnalysis package (Michaud-Agrawal et al., 2011). The proteins were divided into various segments, such as the different TM domains. From each frame of the molecular dynamics simulation, the center of mass coordinates for each segment were extracted. Coordinates were filtered using a gaussian filter and the Euclidean distance between the center of masses was calculated. Slices with the width 5 Å of the TM segments were extracted by defining a plane that passes through the center of masses and choosing the residues distanced 5 Å or less from the plane. The slices were visualized using NGLview (Nguyen et al., 2018). The protein–protein interactions in the MD simulation were analyzed using Schrödinger Suite v2022-2 script analyze_trajectory_ppi.py (Schrödinger Suite 2022-2, Schrödinger, LLC, New York, NY, 2022). The interactions were visualized for the mutated residue based on bond types over time using a custom-written Python script.

### Plasmids and reagents

GB1b, GB2 and SRE-FLuc plasmids were as described (Cediel et al., 2022). GB2 variant constructs were generated from the human Myc-tagged GB2 with the Q5 Site-Directed Mutagenesis kit (New England Biolabs, Ipswich, USA) using the following primers (small caps indicate the mutation): R212Q_F TCTGAGGTGCaGAATGACCTG, R212Q_R GAACCTCTGAACGTCTTG, R212W_F CTCTGAGGTGtggAATGACCTGA, R212W_R AACCTCTGAACGTCTTGC. The following antibodies were used: anti-Myc (#9160, Abcam, Cambridge, United Kingdom; sc-40, Santa Cruz Biotechnology, Dallas, USA), anti-GB2 (#322205, Synaptic systems, Göttingen, Germany), anti-βactin (#4970, Cell Signalling, Danvers, USA), anti-Rb AF488 (#A11008, Invitrogen, Waltham, United States) and anti-Gp AF647 (#A21450, Invitrogen, Waltham, United States). Poly-L-Lysine (#P1399) and GABA (#0344) were from Sigma (Burlington, USA) and Tocris Bioscience (Bristol, England), respectively. Lipofectamine 2000 and SuperSignal West Femto Chemiluminescent Substrate (#34095) was purchased from Thermo Fisher Scientific (Waltham, USA).

### GB2 expression

For imaging and cytochemistry experiments, HEK293T cells were transiently transfected with equal amounts of Myc-GB2 and GB1b plasmids using Lipofectamine 2000. After 6 h, cells were detached and seeded onto Poly-L-Lysine coated glass coverslips in a 24-well plate at a density of 45,000 cells/well. Two days later, the culture medium was replaced with Opti-MEM™-GlutaMAX™ (#31985062; Gibco, Invitrogen, Waltham, United States) supplemented with anti-Myc antibody (1:2000) and cells incubated at 4°C for 15 min. Subsequently, cells were fixed (4% PFA + 4% sucrose, 10 min, room temperature), permeabilized (PBS + 0.2% Triton X-100; 10 min, room temperature) and incubated for 2 h at room temperature with the anti-GB2 antibody (1:500). After incubation, cells were washed three times with 1 × PBS and incubated for 90 min at room temperature with AF488- (Myc) or AF647-conjugated (GB2) secondary antibodies (1:500). Imaging was with Zeiss LSM880 and LSM700 confocal microscopes equipped with PLAN APO 63x oil immersion objectives. Samples were excited with lasers at 488 and 647 nm. Image analysis was conducted with Fiji (ImageJ). Maximum projection images were generated from Z-stacks, and the average fluorescence intensity was measured within regions of interest (ROIs) for individual cells. ROIs of the same shape were used to obtain background values. Myc-to-GB2 fluorescence intensity ratios were calculated from raw fluorescence intensity values for each ROI. Data were tested for normality, and the unpaired t-test with Welch’s correction (GB2^R212Q^) and Mann–Whitney (GB2^R212W^) were used to test for statistically significant differences. For assessing total expression of GB2 on immunoblots, HEK293T cells were lysed in the NETN lysis buffer (100 mM NaCl, 20 mM Tris-Cl pH 8.0, 0.5 mM EDTA, 0.5%(v/v) NP-40) for 20 min at 4°C and centrifuged in a tabletop centrifuge at maximum speed for 15 min. For immunoblot analysis after SDS-PAGE, samples were probed with anti-Myc (Santa Cruz) and anti-βactin antibodies. Immunoblots were developed with SuperSignal West Femto Chemiluminescent Substrate and analysed with Fiji. GB2-to-βactin ratios were calculated and differences in protein expression tested for normality with the Shapiro–Wilk test and for statistical significance with the unpaired *t*-test. Statistical analysis was performed using GraphPad Prism 8. A significance threshold of 0.05 was set for the *p*-value.

### SRE-luciferase assay

HEK293T cells stably expressing Gα_qi_ were transiently transfected with GB1b, GB2 and SRE-FLuc. In experiments designed to model the heterozygous patient situation, WT and variant GB2 cDNA were co-transfected in a 1:1 ratio, and the total amount of transfected cDNA was kept constant for all conditions. Six hours after transfection, cells were distributed into 96-well microplates (Greiner Bio-One) at a density of 70,000 cells/well. After 24 h, the culture medium was replaced with Opti-MEM™-GlutaMAX™. GABA_B_Rs were activated with various concentrations of GABA for 6 h. FLuc activity in lysed cells was measured using the Dual-Luciferase® Assay Kit (Promega) using a Spark® microplate reader (Tecan). Luminescence signals were adjusted by subtracting the luminescence obtained when expressing SRE-FLuc in the absence of GABA_B_Rs. Data were analyzed and plotted in GraphPad Prism 8. The parameters (constitutive activity, Emax, and EC50) of curves fitted to individual experiments were tested for normality with the Shapiro–Wilk test and for statistical significance with one-way ANOVA and Kruskal-Wallis with appropriate post-hoc tests (Tukey’s or Dunnett’s T3 for ANOVA and Dunn’s for Kruskal-Wallis) in GraphPad Prism 8. A significance threshold of 0.05 was set for the *p*-value.

## Results

**Figure 1:**
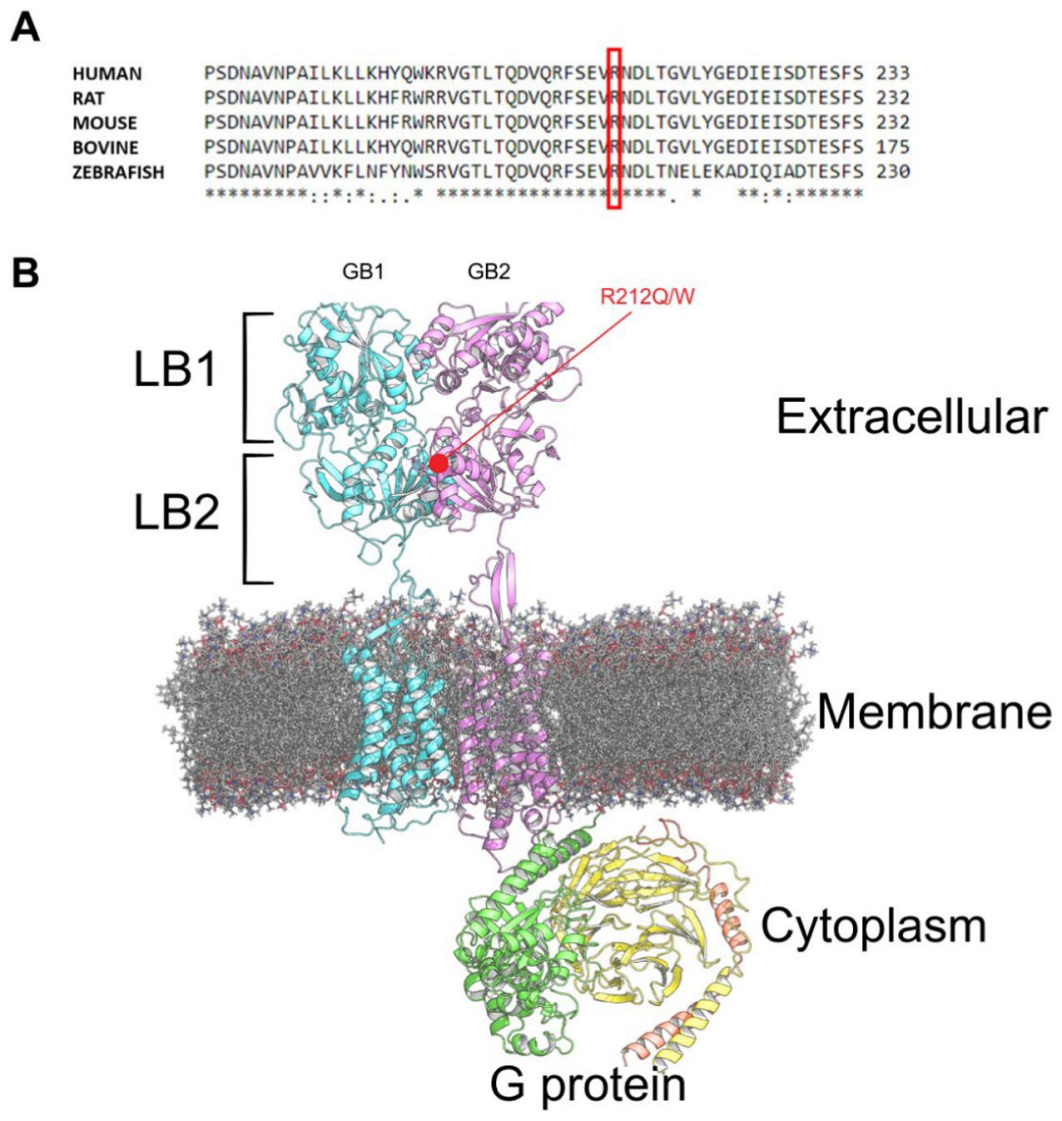
GABA_B_R structure. **(A)** Multiple sequence alignment showing the conserved Arginine residue at position 212 (highlighted in red) across various species. **(B)** GABA_B_R (pdb:7 EB2) bound to G protein embedded in a phospholipid membrane, with the GB2 variants highlighted in red.

We performed MD simulation and functional analysis on two missense variants located in the same site within the *GABBR2* gene. The first variant, p.Arg212Gln, was found on exome sequencing of our patient with autistic spectrum disorder and was absent in parents. This variant has not been previously reported in any database. The second variant, p.Arg212Trp, is located in the same position and was previously reported in gnomAD (see footnote 1), but no clinical data was provided. Both variants are classified as VUS according to the ACMG guidelines. These variants occurred in an evolutionarily conserved position (Figure 1A) located in the GB2 VFTD of the extracellular domain of the receptor. They are positioned within the LB2 domain near the interface that binds to the LB2 domain of GB1 (Figure 1B).

### Influences of *GABBR2* p.Arg212Gln and p.Arg212Trp on the LB2-LB2 receptor interface

**Figure 2:**
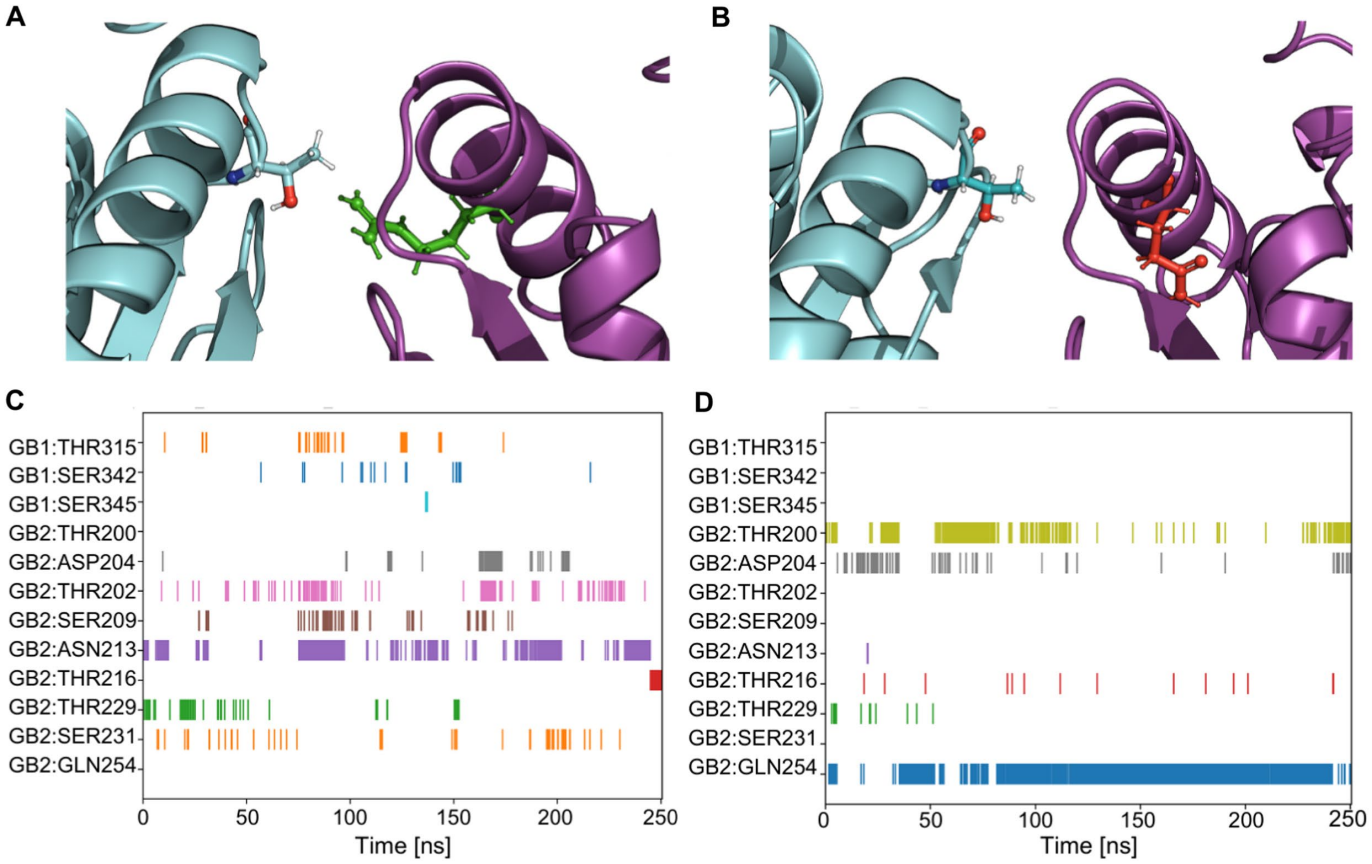
Quantification of amino acid bonds over time for *GABBR2* p.Arg212Gln in the inactive state (pdb:7C7S). **(A)** The GB2 protein, carrying Arg in position 212 is shown in green. The Arg residue faces the GB1 subunit and interacts with it (see quantification in **C**). **(B)** The *GABBR2* p.Arg212Gln variant, carrying a Gln in position 212, is shown in red. The Gln residue faces the GB2 subunit and does not interact with amino acids of GB1 (see quantification in **D**). **(C)** Side chain to side chain bonds occurrence over the molecular dynamics simulation in the GB2 protein. Each row represents a residue that forms bonds with the Arg at position 212. **(D)** Side chain to side chain bonds occurrence over the simulation in the *GABBR2* p.Arg212Gln protein. Each row represents a residue that forms bonds with Gln at position 212.

Homology modeling was performed on the active (pdb:7 EB2) and the inactive (pdb:7C7S) structures of GABA_B_Rs to create structures for the variants. MD simulations were performed with both WT and variant GB2 subunits, in the active and inactive configuration. MD analysis revealed that the Arg212 residue is oriented towards the interface of GB2 with GB1, forming stabilizing bonds to GB1 with its side chain, either directly or indirectly with adjacent amino acids (Figures 2A,C). With the Arg212Gln substitution, a less hydrophilic amino acid, there are no bonds with the GB1 subunit, as its side chain is oriented towards GB2 (Figures 2B,D). With the Arg212Trp substitution, a hydrophobic amino acid, Trp212 had only one side chain bond with Thr200 in GB2 and no bonds with the GB1 subunit (Supplementary Figure S1).

**Figure 3:**
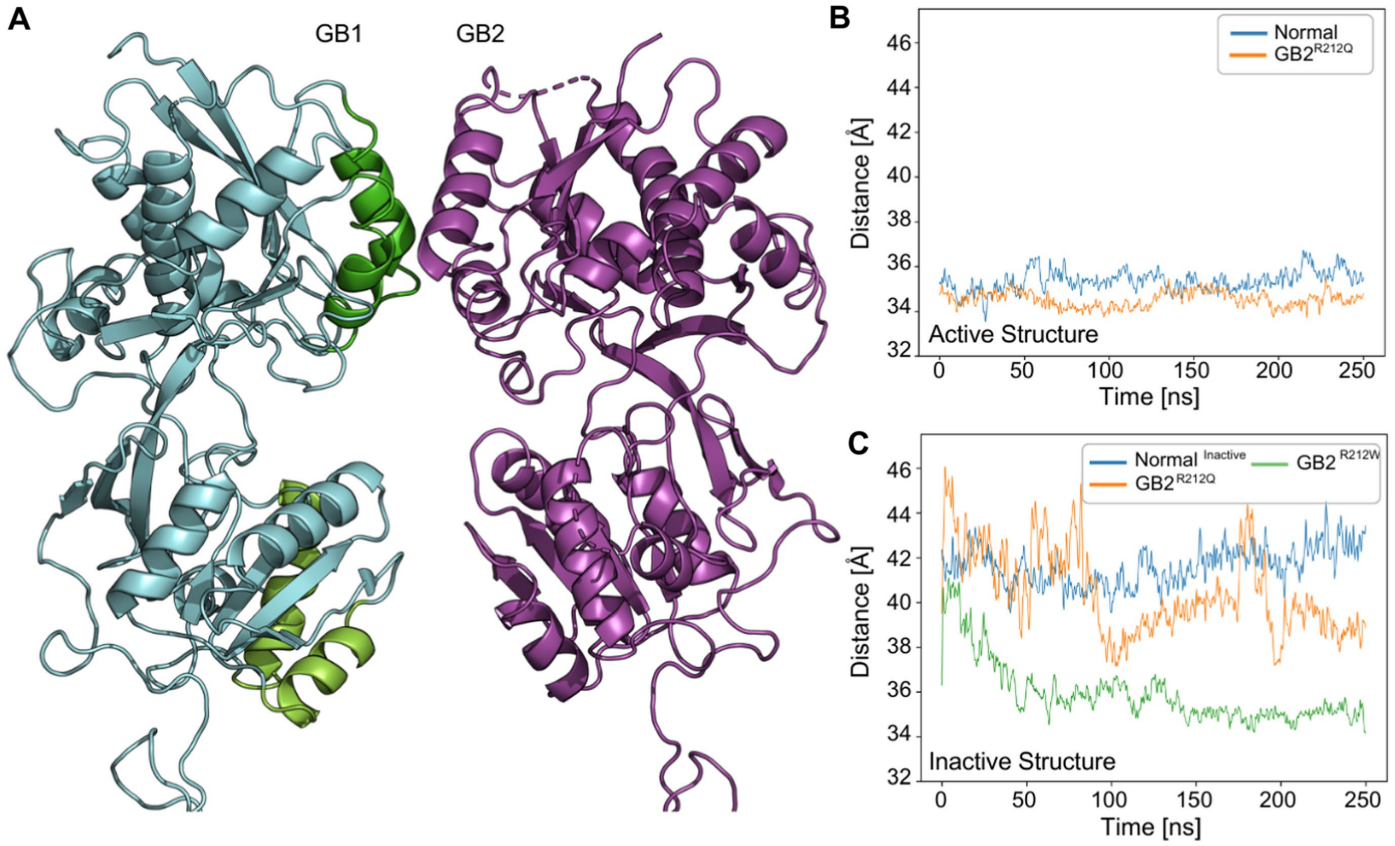
VFTD dynamics of *GABBR2* p.Arg212Gln and p.Arg212Trp in active and/or inactive states (pdb:7 EB2,7C7S). **(A)** Illustration of the inactive structure (pdb: 7C7S) of *GABBR2* p.Arg212Gln. We monitored the distance between GB1-LBupper (the center of mass of Cα for residues 222–235 and 247–260; green) and GB1-LBlower (the center of mass of Cα for residues 347–358 and 368–382; light green). **(B)** Distance in angstrom between GB1-LBupper and GB1-LBlower in the active structure (pdb:7 EB2) throughout the simulation. p.Arg212Trp was not simulated in the active structure. **(C)** The distance between GB1-LBupper and GB1-LBlower in the inactive structure (pdb: 7C7S). *GABBR2* p.Arg212Gln and p.Arg212Trp exhibit lower values than those of the WT receptor, closer to those observed in the active structure of the WT receptor.

Yang et al. (2022) measured the distance within the GB1 VFTD during activation. They found that the LB1-LB2 [“LBupper” and “LBlower” in Yang et al. (2022); Figure 3A] distance is approximately 41 Å before activation, whereas in the closed active conformation, it is around 33 Å (Yang et al., 2022). In our simulations, when we measured the LB1-LB2 distance in GB1, we observed that the active (closed) conformation (pdb:7 EB2) exhibited distances of about 34–35 Å for both GB2 and p.Arg212Gln (Figure 3B). In the case of the inactive (open) conformation, the measurements for this distance with GB2 were approximately 41–42 Å, while with p.Arg212Gln lower values of 37.5 Å were obtained. These values for the inactive conformation of the receptor incorporating p.Arg212Gln were closer to those of the active conformation, suggesting that the variant receptor displays increased constitutive activity (Figure 3C).

Next, we measured the LB1-LB2 distance for p.Arg212Trp in the inactive (open) conformation. Surprisingly, the values were with 34–35 Å similar to those measured in the active (closed) conformation for GB2 (Figure 3C). These results suggest that receptors incorporating p.Arg212Trp exhibit increased constitutive activity compared to receptors incorporating p.Arg212Gln. However, this prediction contradicts the TM intersubunit interface prediction for p.Arg212Trp (see below).

### Influences of *GABBR2* p.Arg212Gln and p.Arg212Trp on the TM intersubunit interfaces

To further elucidate the effects of the structural changes on the functionality of the receptor, we examined the TM domains using MD. Previous studies demonstrated that upon activation of GABA_B_Rs, the TM3-TM5/TM3-TM5 interface in the inactive state transitions to a TM6/TM6 interface in the active state (Kim et al., 2020; Mao et al., 2020; Shaye et al., 2020). Additionally, TM4s and TM5s move apart during the activation process. Specifically, Kim et al. (2020) showed that in the inactive state, GABA binding forces the extracellular domains of GB1 and GB2 into a compact form, relocating the linkers that connect the extracellular and 7TMDs and bringing them closer together. The movement of the linker, along with the associated extracellular loop 2 of the 7TMD, reorients the two 7TMDs and establishes a new interface involving the TM5, TM6 and TM7 helices (Kim et al., 2020).

**Figure 4:**
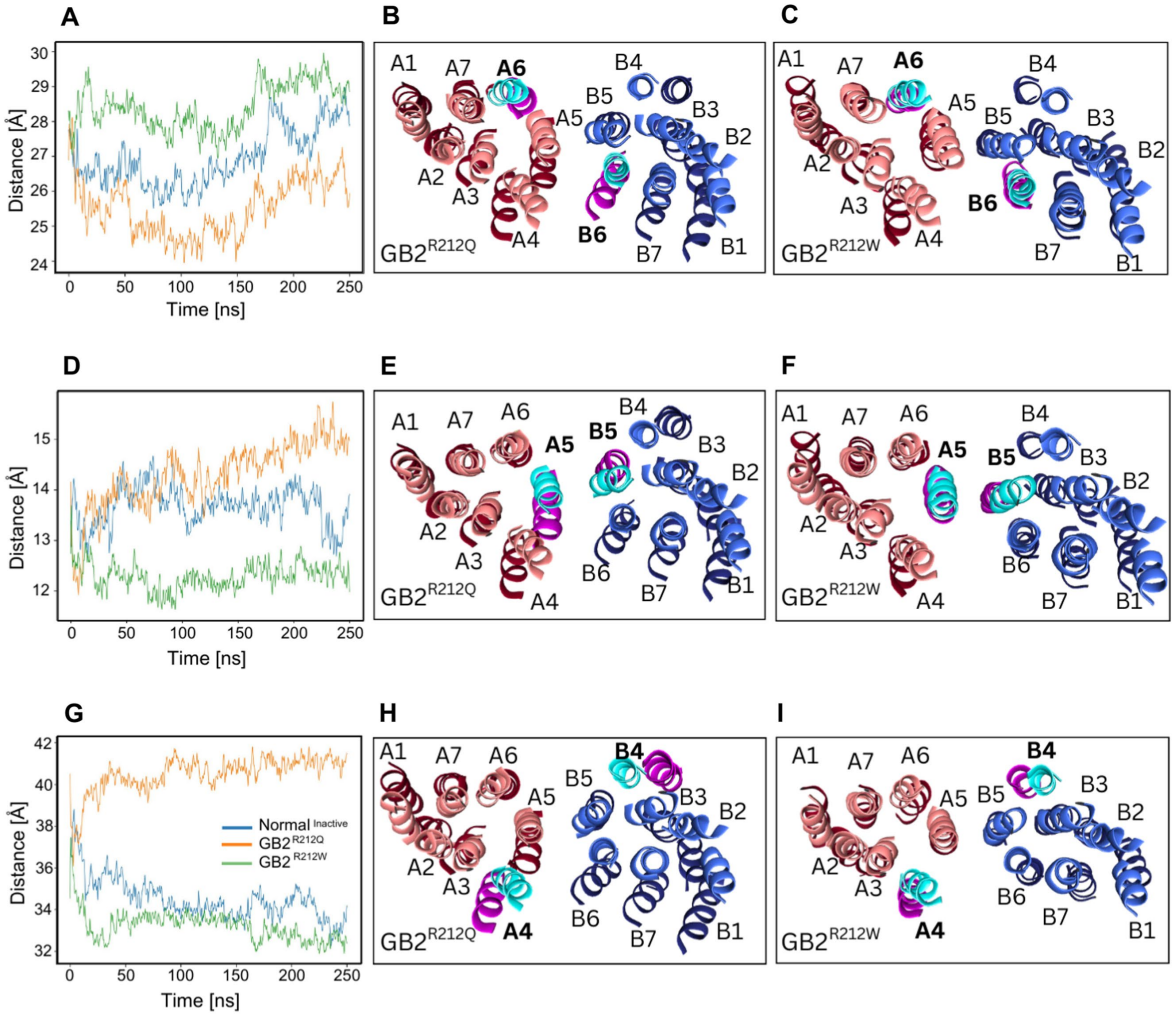
*GABBR2* p.Arg212Gln and p.Arg212Trp TM segment distances. **(A)** The distance between centers of mass of TM6 helices (805–825 in GB1, 692–712 in GB2) segments over time. **(B,C)** Illustration demonstrating that TM6 helices are closer together in the inactive structure of p.Arg212Gln than in WT receptor, while TM6 helices are further apart in p.Arg212Trp than in WT receptor. **(D)** The distance between centers of mass of TM5 helices (769–789 in GB1, 655–675 in GB2) segments over time. **(E,F)** In the inactive structures of p.Arg212Gln receptors, TM5 helices exhibit a greater separation compared to their positioning in WT receptors. Conversely, in p.Arg212Trp receptors, TM5 helices are observed to be closer together than in the WT receptor structures. **(G)** The distance between centers of mass of TM4 helices (711–731 in GB1, 598–618 in GB2) segments over time. **(H,I)** In the inactive structure of the WT receptor, TM4 helices are closer together than in the inactive structure of the p.Arg212Gln variant. The values for p.Arg212Trp closely resemble those observed for the WT receptor. Additional distance comparisons for TM1-3 and TM7 are shown in Supplementary Figure S2. GB1 is in red, GB2 is in blue. Darker shades represent GB2 variants. Lighter shades represent the GB2 protein. The GB2 protein segment is shown in cyan, and the *GABBR2* p.Arg212Gln\Trp segment in pink.

To assess the level of receptor activity using MD simulations, we measured the distances between the TM domains in the inactive structure (pdb:7C7S) after removing the antagonist CGP54626. Our simulations revealed that the intersubunit distance of TM6 was reduced in the presence of GB2^R212Q^ when compared to GB2, while it was increased in the presence of GB2^R212W^ (Figures 4A–C). Additionally, we observed an increased TM5 intersubunit distance with GB2^R212Q^ and a decreased TM5 intersubunit distance with GB2^R212W^ (Figures 4D–F). Surprisingly, the TM4 intersubunit distance was found to be larger in the presence of GB2^R212Q^ while in GB2^R212W^ it is similar to GB2 (Figures 4G–I). Additional comparisons of TM distances for TM1-3 and TM7 did not demonstrate considerable differences (Supplementary Figure S2). In the case of GB2^R212Q^, the structural alterations observed in both the VFTDs and the TM domains are consistent and support a bias towards an active conformation. However, for GB2^R212W^, the structural alterations in the VFTDs and TM domains are inconsistent. As a result, we performed *in vitro* functional assays in order to evaluate the predictability of our MD simulations.

### Functional characterization of GABA_B_Rs assembled with GB2^R212Q^ and GB2^R212W^

**Figure 5:**
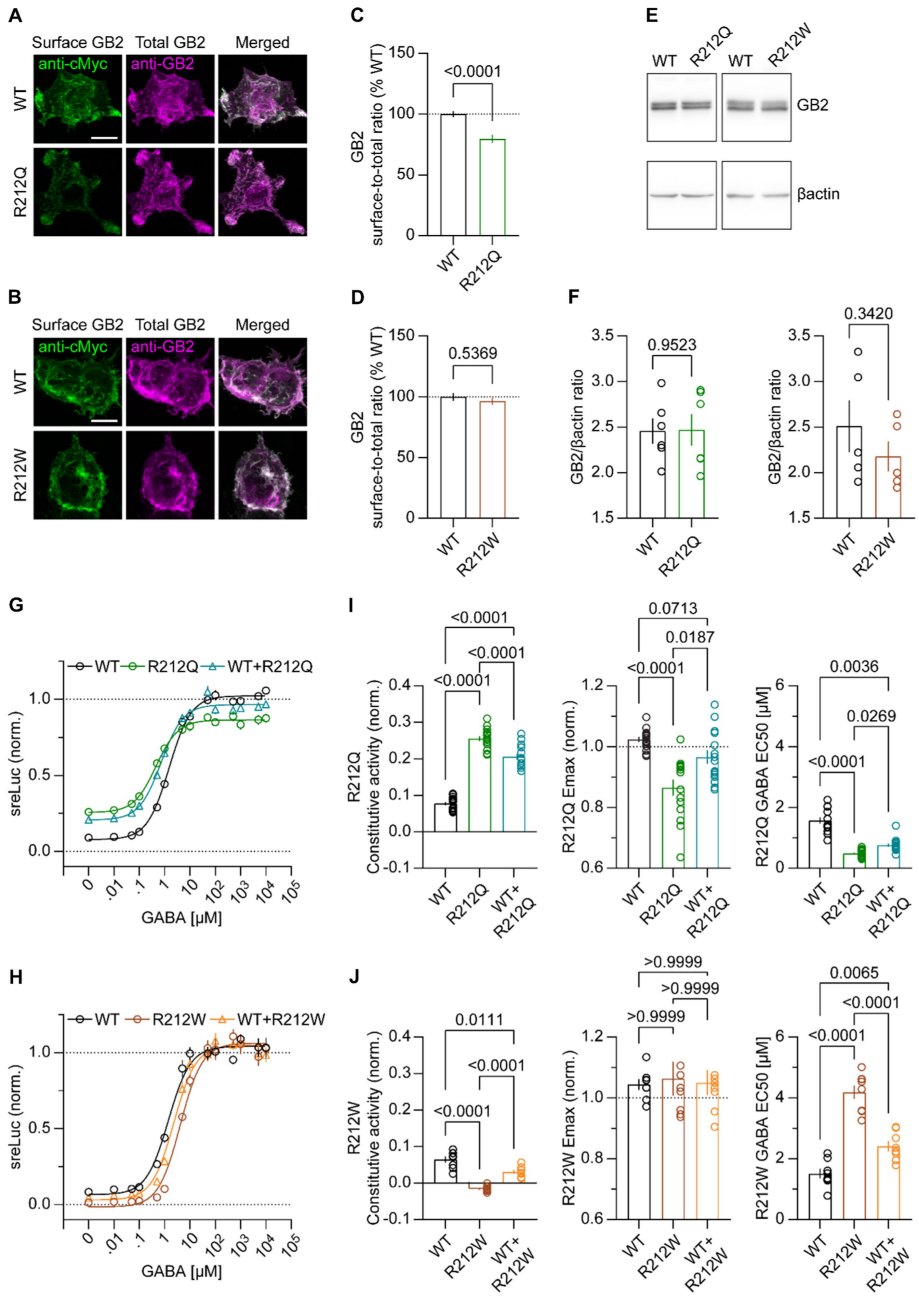
Functional characterization of GB1/2^R212Q^ and GB1/2^R212W^ receptors in heterologous cells. **(A,B)** Cell surface and total Myc-GB2 expression of GB1/2, GB1/2^R212Q^ **(A)** and GB1/2^R212W^ **(B)** receptors in transfected HEK293T cells. GB1 and Myc-GB2 subunits were co-expressed and the surface GB2 FIGURE 5 (Continued)immunofluorescence assessed in living cells using anti-Myc and AF488-conjugated secondary antibodies. Total GB2 immunofluorescence was determined with anti-GB2 and secondary AF647-conjugated antibodies after cell permeabilization. Scale bar: 10 μm. **(C,D)** Bar graphs showing the GB2, GB1/2^R212Q^ **(C)** and GB1/2^R212W^ **(D)** surface-to-total immunofluorescence ratios. GB1/2 (100.00 ± 2.42% *n* = 78), GB1/2^R212Q^ (79.70 ± 3.25% *n* = 84), unpaired *t*-test with Welch’s correction; GB1/2 (100 ± 3.11% *n* = 128), GB1/2^R212W^ (96.42 ± 3.03% *n* = 107), Mann–Whitney test. **(E)** Representative immunoblots of HEK293T cells expressing GB1/2, GB1/2^R212Q^, and GB1/2^R212W^. Myc-GB2 was detected with the anti-Myc antibody. **(F)** Total expression of GB1/2, GB1/2^R212Q^, and GB1/2^R212W^ in HEK293T cells. GB1/2 (2.46 ± 0.14 *n* = 6), GB1/2^R212Q^ (2.47 ± 0.17 *n* = 6); GB1/2 (2.51 ± 0.28 *n* = 5); GB1/2^R212W^ (2.18 ± 0.17 *n* = 5), unpaired t-test. **(G)** Concentration-response curves depicting GABA-induced luciferase activity (sreLuc bioluminescence) of GB1/2 and GBR1/2^R212Q^ in transfected HEK293T cells. GABA concentration-response curves were fitted using a three-parameter log (agonist) vs. response nonlinear regression curve. n = 15 experiments for each curve. **(H)** Same as in (G) for GBR1/2^R212W^. *n* = 8 experiments for each curve. **(I)** Bar graphs of constitutive activity, Emax and EC50 values for GABA concentration–response curves at GB1/2, GB1/2^R212Q^ and GB1/2 + GB1/2^R212Q^ receptors. ANOVA with Tukey’s post-hoc analysis (constitutive activity, EMax), and Kruskal-Wallis with Dunn’s post-hoc (EC50). **(J)** Same as in **(I)** for GBR1/2^R212W^ receptors. ANOVA with Dunnett’s T3 post-hoc (constitutive activity), Kruskal-Wallis with Dunn’s post-hoc (Emax), and ANOVA with Tukey’s post-hoc (EC50). All data are mean ± SEM. *p*-values are given in the bar graphs.

To address whether GB2 subunit variants alter GABA_B_R activity, we initially addressed whether receptors assembled with GB1 together with Myc-GB2^R212Q^ or Myc-GB2^R212W^ traffic normally to the cell surface. We assessed cell surface levels of GB2 protein in transfected HEK293T cells with an anti-Myc antibody, and total GB2 protein levels after cell permeabilization using an anti-GB2 antibody (Figures 5A,C). GB2 surface expression was quantified as the ratio of surface-to-total immunofluorescence in maximum projection images (Figures 5B,D). The results showed that surface expression of GABA_B_Rs assembled with GB2^R212Q^ was reduced compared to WT receptors, while GB2^R212W^ had no impact on cell-surface expression. Total expression of GB2^R212Q^ and GB2^R212W^ was unchanged compared to GB2 (Figures 5E,F), showing that reduced surface expression of GB2^R212Q^ is not due to receptor degradation. Next, we generated GABA concentration-response curves for both WT and variant GABA_B_Rs using an assay that couples receptors to phospholipase C (PLC) through chimeric Gα_qi_ (Stefan et al., 2007). We monitored PLC activity with a serum responsive element-luciferase (SRE-Luc) reporter. Increasing concentrations of GABA yielded sigmoidal concentration-response curves for WT and variant receptors (Figures 5G,H). GB1/2^R212Q^ receptors exhibited increased constitutive activity (GB1/2 0.08 ± 0.01; GB1/2^R212Q^ 0.26 ± 0.01, *p* < 0.0001 ANOVA), a reduced Emax (GB1/2 1.02 ± 0.01; GB1/2^R212Q^ 0.86 ± 0.03, *p* < 0.0001 ANOVA), and a reduced EC50 for GABA (GB1/2 1.58 ± 0.10 μM; GB1/2^R212Q^ 0.49 ± 0.04 μM, *p* < 0.0001 Kruskal-Wallis; Figure 5I). In contrast, GB1/2^R212W^ receptors exhibited reduced constitutive activity (GB1/2 0.06 ± 0.01; GB1/2^R212W^ -0.02 ± 0.01, *p* < 0.0001 ANOVA), an increased EC50 (GB1/2 1.51 ± 0.16 μM; GB1/2^R212W^ 4.18 ± 0.22 μM, *p* < 0.0001 ANOVA), and a normal Emax (GB1/2 1.04 ± 0.02; GB1/2^R212W^ 1.06 ± 0.06, *p* = 0.8817 Kruskal-Wallis; Figure 5J). As affected individuals are heterozygous for the *GABBR2* variants, we co-expressed variant and WT GB1/2 receptors and determined the concentration-response curves of the mixed receptor population (Figures 5G,H). The mixed GB1/2 and GB1/2^R212Q^ receptor population exhibits increased constitutive activity (0.21 ± 0.01) and decreased EC50 (0.76 ± 0.05 μM) compared to GB1/2 receptors, while the Emax is not significantly different from that of GB1/2 receptors (0.97 ± 0.02). A mixed GB1/2 and GB1/2^R212W^ receptor population exhibits decreased constitutive activity (0.03 ± 0.01) and increased EC50 (2.40 ± 0.17 μM) compared to GB1/2 receptors. Overall, the functional assay results indicate that GB2^R212Q^ receptors exhibit induce a gain-of-function, while GB2^R212W^ receptors exhibit induce a loss-of-function, as evidenced by their constitutive activity and EC50 values. However, the reduced Emax observed with GB1/2^R212Q^ receptors could potentially oppose to a gain-of-function *in vivo*.

## Discussion

This illustrative case highlights a critical requirement in modern medicine, particularly when encountering VUS identified through genomic or exomic sequence data. Predicting the pathogenicity of a variant and understanding how it affects protein function are crucial for accurate diagnosis and effective treatment. Traditional bioinformatic platforms are commonly employed in deep sequencing pipelines to classify genetic variants. Popular computational tools like SIFT,^3^ MutationAssessor,^4^ PolyPhen-2,^5^ Condel,^6^ and PROVEAN^7^ primarily predict the likelihood of a genetic variant being pathogenic. They do not offer insights into whether a variant leads to a gain or loss-of-function, or the extent of impairment in protein function (Nissenkorn et al., 2019). Rapid development in the field of genomics resulted in a gap between the number of protein variants identified and the number of experimentally validated 3D protein structures. Ideally, functional studies would be conducted for each identified VUS; however, the necessary assays are typically carried out in specialized laboratories and may not be easily accessible to clinicians. Consequently, the development of computerized platforms based on artificial intelligence, such as MD simulations, becomes imperative to bridge this gap. MD simulations employ mathematical equations to determine the atomic positions at each time point, thereby enabling predictions of protein motion and inference with protein function. A major limitation of these mathematical models is the template used for homology modeling of mutant proteins. In this paper, we worked with the known crystal structures of the active and inactive states of GABA_B_Rs. To determine the structure of VUS, we used homology modeling due to the absence of practical alternatives, other than expensive and time-consuming experimental structure determination methods. We optimized the homology model, a common practice in the field, by performing energy minimization using molecular mechanics force fields to mitigate atomic clashes and correct major and minor errors, as described (Hameduh et al., 2020). While the reliability of these predictions is less accurate than experimental structure determination, for individuals carrying VUS, this approach represents the most practical and valuable solution.

Previous studies on *GABBR2* missense variants have revealed a correlation between the phenotype, such as epileptic encephalopathy vs. Rett-like phenotype without seizures, and the type of the variant (gain or loss-of-function), as well as the severity of the dysfunction (Yoo et al., 2017; Vuillaume et al., 2018; Samanta and Zarate, 2019). In this study, we used the MD pipeline to analyze the *GABBR2* p.Arg212Gln variant in a child diagnosed with Level 3 Autism Spectrum Disorder. The objective was to predict whether this previously unreported VUS is disease causing. We established the 3D structure of the protein variant using traditional bioinformatics techniques such as homology modeling. Subsequently, we utilized the Schrödinger algorithm to conduct time-dependent molecular dynamic simulations, which further supported our predictions. Through structural and MD analyses, we discovered that the Gln residue in *GABBR2* p.Arg212Gln does not establish intersubunit bonds with amino acids in GB1 and instead faces the core of GB2. Additionally, we observed a slight closure of the GB1 VFTD and a subtle movement of the TM domain towards an active state arrangement of the helices in the *GABBR2* p.Arg212Gln protein, as compared to the WT protein (Kim et al., 2020). Specifically, our measurements revealed that with the *GABBR2* p.Arg212Gln variant, the TM6 segments are closer together, while the TM5 segments are farther apart, resembling the conformational changes associated with the receptor’s transition from an inactive to an active state. These observations suggest a gain-of-function variant. We additionally found that the TM4 segments are farther apart with the *GABBR2* p.Arg212Gln variant. It was demonstrated by Xue et al. (2019) that during activation TM4 segments move away from one another, which strengthens our assessment that the *GABBR2* p.Arg212Gln variant causes a gain-of-function. We observed in functional assays that GB1/2^R212Q^ receptors indeed exhibit increased constitutive activity and a decreased EC50, consistent with a gain-of-function. However, the increased constitutive receptor activity also diminishes the maximal efficacy of GABA, as observed with other *GABBR2* variants in heterologous assays (Yoo et al., 2017). Moreover, we observed a reduced receptor surface expression in the absence of GB2^R212Q^ protein degradation. Reduced cell surface expression may therefore be attributed to impaired cell surface transport or enhanced internalization of receptors from the plasma membrane. Reduced efficacy of GABA and reduced surface expression likely contribute or cause the diminished EMax in the functional assay system. It is important to note that our MD simulations do not allow us to predict changes in surface expression. When combining WT and GB1/2^R212Q^ receptors in the functional assay system, the mixed receptor population exhibits significantly increased constitutive activity and decreased EC50 compared to WT receptors, while the Emax is not significantly different from that of WT receptors. It is challenging to translate these *in vitro* findings to the *in vivo* situation, but these observations suggest that in the patient, the gain-of-function effect of the variant may be more pronounced than the loss-of-function effect on the Emax. However, it is conceivable that heightened constitutive activity in the patient leads to adaptive changes, e.g., a further receptor downregulation that results in an overall loss-of-function.

Increased constitutive activity caused by the *GABBR2* p.Arg212Gln may cause a shift in the excitation/inhibition balance in the brain towards excess inhibition (Rondard et al., 2008; Geng et al., 2013). The use of a GABA_B_R inverse agonist/antagonist may, therefore, be considered as a treatment option. GABA_B_R antagonists are not readily available as FDA or EMA approved drugs. However, there is limited experience from phase 2 trials involving the compound SGS-742, a low-affinity GABA_B_R antagonist. These trials have been conducted in adults with mild cognitive impairment (Froestl et al., 2004) and children with succinic semialdehyde dehydrogenase deficiency (Schreiber et al., 2021). For patients with gain-of-function variants in GABA_B_Rs, the utilization of SGS-742 or similar compounds would represent a personalized medicine approach.

The *GABBR2* p.Arg212Gln variant affects the same position as the previously reported *GABBR2* p.Arg212Trp variant (gnomAD: 9-101258793-G-A), for which no clinical features have been reported. This latter variant is predicted by Polyphen and SIFT tools to have a deleterious impact on receptor function [gnomAD SNV:9-101258793-G-A (GRCh37)]. Our computational analysis revealed that the p.Arg212Trp alteration shifts the conformation of the VFTD of GB1 towards the active state. In contrast, we measured a decreased distance between the TM5 domains of GB1 and GB2, and an increased distance between the TM6 domains, which suggests a conformational shift towards the inactive state (Shaye et al., 2021). The functional assay with GB1/2^R212W^ receptors demonstrated a loss of function, indicating that changes in TM distances in MD simulations are more reliable indicators of activity alterations than changes observed in the VFTDs. Our MD simulations indicate that this variant exhibits fewer GB1-GB2 intersubunit interactions, suggesting an impact on the overall interaction between these subunits. Moreover, a recent study showed bending and twisting of the VFTDs in the inactive and active states during an 800 nanoseconds simulation of the normal GABA_B_R (Shaye et al., 2020). Therefore, it is possible that the decreased intersubunit interactions and the natural bending of the VFTDs led the VFTDs to arrange in a conformation similar to the active one but with no functional impact as our functional assay show. We conclude that for our shorter simulations lasting 250 nanoseconds, TM distances serve as the most reliable criterion for predicting a gain or loss-of-function in GABA_B_Rs.

Based on the combined computational and functional data, we propose that the *GABBR2* p.Arg212Gln variant is pathogenic. Our findings align with the clinical presentation of the patient, who displayed autistic Rett-like features but no epilepsy. Interestingly, a similar Rett-like phenotype without epilepsy has been reported, which was associated with a missense variant in *GABBR2* leading to elevated constitutive receptor activity (Vuillaume et al., 2018). In summary, our study highlights the potential of computational methods and molecular dynamics simulations to assist physicians in determining whether a receptor variant induces gain- or loss-of-function, thereby guiding personalized treatment for patients.

## Data availability statement

The original contributions presented in the study are publicly available. This data can be found here: https://www.ncbi.nlm.nih.gov/clinvar/variation/985861/ accession number VCV000985861.2.

## Ethics statement

Written informed consent was obtained from the minor(s)’ legal guardian/next of kin for the publication of any potentially identifiable images or data included in this article.

## Author contributions

NB: Conceptualization, Data curation, Formal Analysis, Investigation, Writing – original draft, methodology. MS: Data curation, conceptualization, methodology, investigation and formal analysis, Writing – original draft. IR: Formal Analysis, Methodology, Visualization, Investigation. KF: Visualization, Writing – review & editing. SW-A: Conceptualization, Methodology, Supervision, Project administration. SF: Conceptualization, methodology, investigation and formal analysis, Writing – review & editing. BB: Funding acquisition, Conceptualization, Project administration, Writing – review & editing, Supervision. AN: Data curation, Project administration, Writing – review & editing, Supervision.

## Footnotes

^1^https://gnomad.broadinstitute.org/

^2^https://opm.phar.umich.edu/

^3^http://sift.jcvi.org

^4^http://mutationassessor.org

^5^http://genetics.bwh.harvard.edu/pph2

^6^http://bbglab.irbbarcelona.org/fannsdb

^7^http://provean.jcvi.org

## Supplementary material

SUPPLEMENTARY FIGURE S1 Quantification of amino acid bonds over time for the *GABBR2* p.Arg212Trp variant in the inactive structure (pdb:7C7S). **(A)** Side chain to side chain bonds occurrence over the molecular dynamics simulation in the WT receptors. Each row represents a residue that forms bonds with Arg in position 212. **(B)** Side chain to side chain bonds occurrence over the simulation in the *GABBR2* p.Arg212Trp variant. Each row represents a residue that forms bonds with Trp in position 212.

SUPPLEMENTARY FIGURE S2 TM segment distances in the *GABBR2* p.Arg212Gln and p.Arg212Trp compared to the WT receptor. **(A)** The distance between centers of mass of TM1 helices (592-612 in GB1; 484-504 in GB2) segments over time. **(B)** The distance between centers of mass of TM2 helices (632-652 in GB1; 523-543 in GB2) segments over time. **(C)** The distance between centers of mass of TM3 helices (668-688 in GB1; 552-572 in GB2) segments over time. **(D)** The distance between centers of mass of TM7 helices (834-854 in GB1; 721-741 in GB2) segments over time.
