# Supplementary figures and images for: Characterization of a de novo GABBR2 variant linked to autism spectrum disorder
Source: Front Mol Neurosci. 2023 Nov 23;16:1267343. doi: 10.3389/fnmol.2023.1267343 (PMC10710151; doi:10.3389/fnmol.2023.1267343)

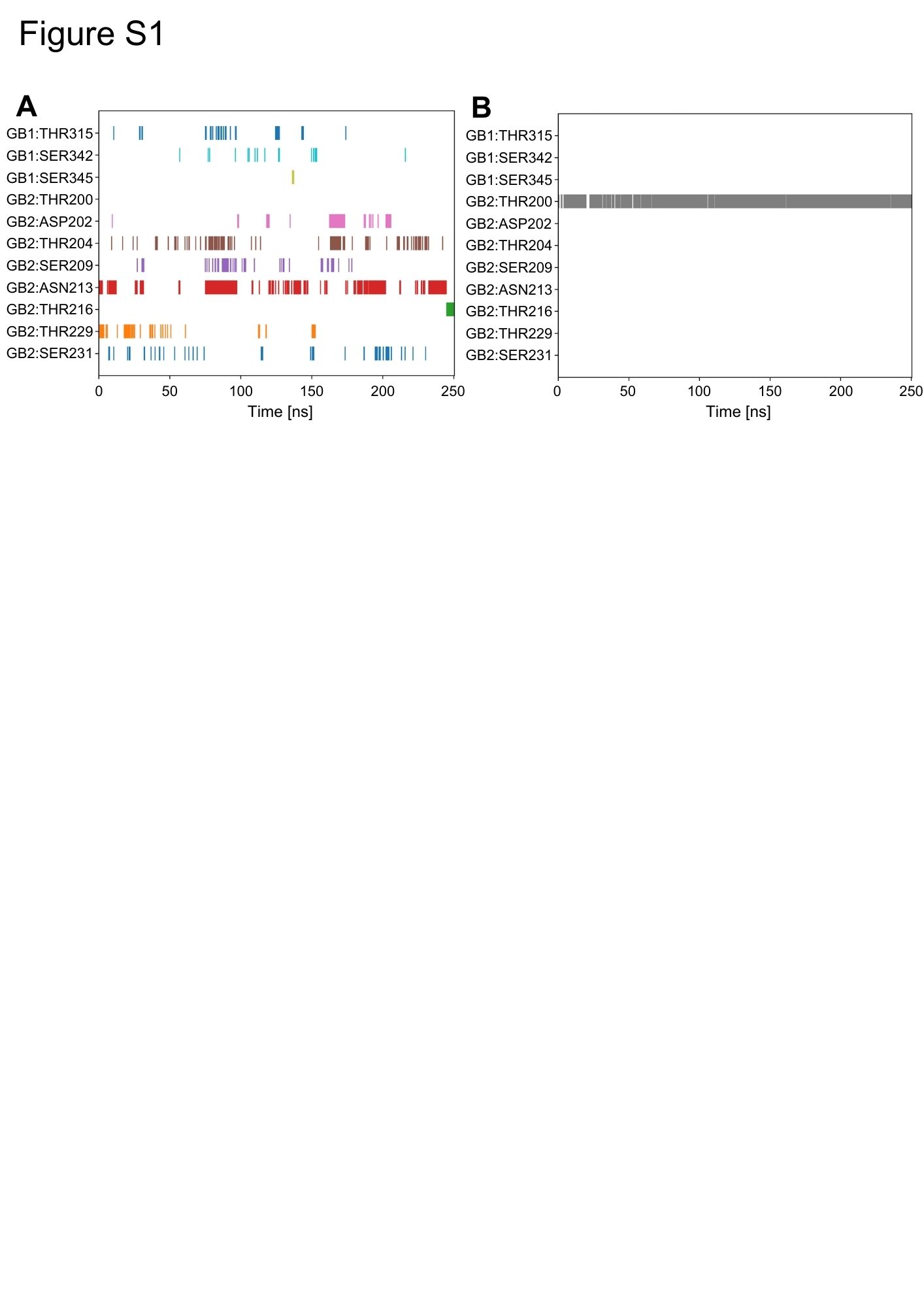

Supplement: SUPPLEMENTARY FIGURE S1 — Quantification of amino acid bonds over time for the GABBR2 p.Arg212Trp variant in the inactive structure (pdb:7C7S). (A) Side chain to side chain bonds occurrence over the molecular dynamics simulation in the WT receptors. Each row represents a residue that forms bonds with Arg in position 212. (B) Side chain to side chain bonds occurrence over the simulation in the GABBR2 p.Arg212Trp variant. Each row represents a residue that forms bonds with Trp in position 212. [file Image_1.JPEG]

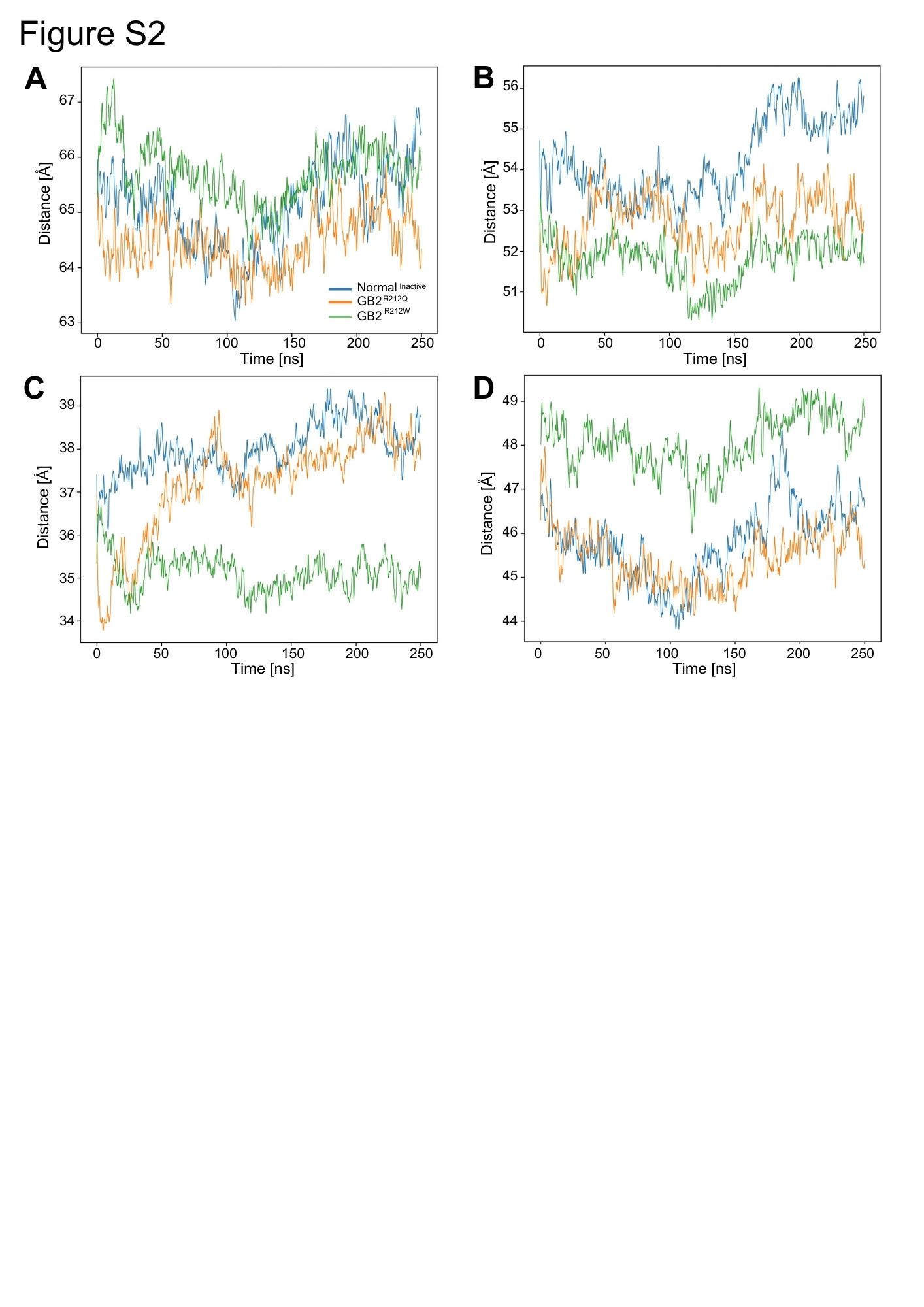

Supplement: SUPPLEMENTARY FIGURE S2 — TM segment distances in the GABBR2 p.Arg212Gln and p.Arg212Trp compared to the WT receptor. (A) The distance between centers of mass of TM1 helices (592-612 in GB1; 484-504 in GB2) segments over time. (B) The distance between centers of mass of TM2 helices (632-652 in GB1; 523-543 in GB2) segments over time. (C) The distance between centers of mass of TM3 helices (668-688 in GB1; 552-572 in GB2) segments over time. (D) The distance between centers of mass of TM7 helices (834-854 in GB1; 721-741 in GB2) segments over time. [file Image_2.JPEG]
